# 3p14 De Novo Interstitial Microdeletion in a Patient with Intellectual Disability and Autistic Features with Language Impairment: A Comparison with Similar Cases

**DOI:** 10.1155/2015/876348

**Published:** 2015-05-14

**Authors:** Ana Belén de la Hoz, Hiart Maortua, Ainhoa García-Rives, María Jesús Martínez-González, Maitane Ezquerra, María-Isabel Tejada

**Affiliations:** ^1^Plataforma de Genética Genómica, Instituto de Investigación Sanitaria BioCruces (IIS BioCruces), Hospital Universitario Cruces, Barakaldo, 48903 Bizkaia, Spain; ^2^GCV-CIBER de Enfermedades Raras (CIBERER-ISCIII), 28029 Madrid, Spain; ^3^Laboratorio de Genética Molecular, Servicio de Genética, IIS BioCruces, Hospital Universitario Cruces, Barakaldo, 48903 Bizkaia, Spain; ^4^Sección de Neuropediatría del Servicio de Pediatría, IIS BioCruces, Hospital Universitario Cruces, Barakaldo, 48903 Bizkaia, Spain

## Abstract

To date, few cases of 3p proximal interstitial deletions have been reported and the phenotype and genotype correlation is not well understood. Here, we report a new case of a 3p proximal interstitial deletion. The patient is an 11-year-old female with speech and social interaction difficulties, learning disability, and slight facial dysmorphism, but no other major malformations. An 8 Mb de novo interstitial deletion at 3p14.2-p14.1, from position 60.461.316 to 68.515.453, was revealed by means of array comparative genomic hybridization and confirmed using quantitative reverse-transcription polymerase chain reaction assays. This region includes six genes: *FEZF2, CADPS, SYNPR, ATXN7, PRICKLE*, and *MAGI1*, that are known to have a role in neurodevelopment. These genes are located on the proximal side of the deletion. We compare our case with previously well-defined patients reported in the literature and databases.

## 1. Introduction

It has previously been reported that interstitial deletions of chromosome 3p are rather rare and there are no well-defined breakpoints. However, since the first report of this condition in 1979 by Kogame and Kudo [1], various heterozygous overlapping deletions involving the short arm of chromosome 3 have been found in patients with global developmental delay, intellectual disability, language impairment, and autistic features, but without any other major malformations [2–6]. Some other features have also been correlated with this alteration, namely, defective lymphopoiesis [7] and defective cardiac development [8]. All of the aforementioned authors believed that* FOXP1* was responsible for these features.

Recently, various authors [9–11] have published studies in which they characterized proximal deletions in 3p in a total of six patients, accurately defining their phenotypical features. None of those overlapping deletions affected the* FOXP1* gene. All of the patients had intellectual disabilities, gross motor delay, slight facial dysmorphism, nonexpressive language, and autistic features. After comparing their patients with four individuals with 3p14-deletions reported with full clinical descriptions in the Database of Chromosomal Imbalance and Phenotype in Humans using Ensembl Resources (DECIPHER) database, they concluded that all these deletions in 3p14 were associated with very similar features, namely, intellectual disability, autistic features, developmental delay, and often speech impairment but only mild facial dysmorphism. The lack of external features characteristic in these patients makes it difficult to reach a correct diagnosis without array CGH analysis. In addition, the shortage of cases in the literature makes it difficult to identify the gene or the core region responsible for these phenotypes. Here, we report a new case of 3p14 deletion, because we consider it extremely important to share information on cases of deletions that involve this genomic region in order to identify the gene or genes associated with these disorders.

## 2. Case Presentation

The child is a female first-born of healthy nonconsanguineous parents. There is no family history of congenital abnormalities or intellectual disability and the pregnancy was unremarkable. She was born at term (39 weeks of gestation) by normal delivery, with a weight of 2400 g (<p3) and head circumference of 33.5 cm (<p25). Her Apgar score was 7/10 at 1 and 5 minutes and there were no remarkable observations in the perinatal period. She was first evaluated by our neuropediatric team at the age of 6 months because of poor response to stimuli and lack of a social smile. Clinical examination revealed significant motor developmental delay; a cranial ultrasound, an electroencephalogram, and auditory evoked potentials testing were requested, with results being normal in all cases.

**Figure 1:**
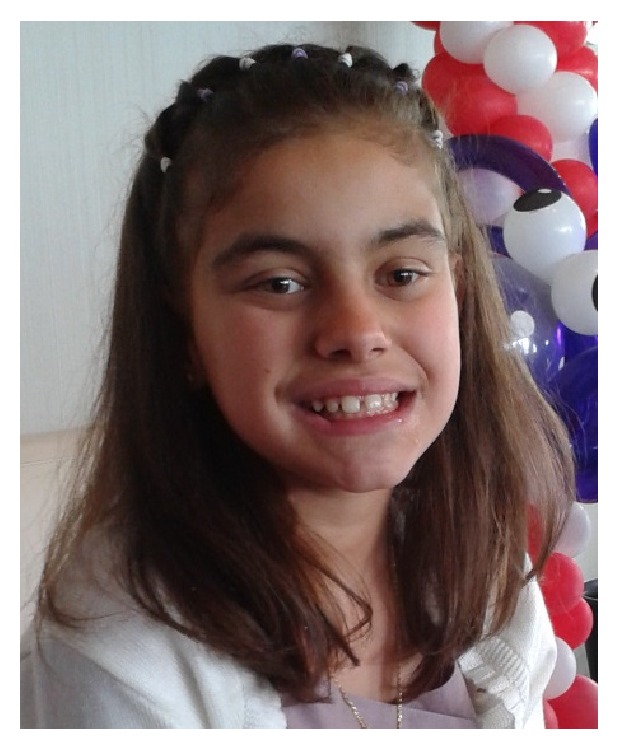
The facial photograph of the patient does not show any remarkable phenotypic features. Only slight facial dysmorphism could be observed: a long face with a prominent chin; broad forehead; and a broad, large mouth with widely spaced upper front teeth. Although not visible in this photograph, she has slightly protruding prominent ears.

During the first year of life, moderate psychomotor impairment became evident and, therefore, from the age of 12 months, she has received cognitive stimulation therapy. At 20 months, despite motor clumsiness, the patient was able to walk without support but had speech difficulties with a marked expressive language disorder and global learning problems. She had some autistic features including stereotypic movements and difficulties with eye contact. In a psychometric assessment at 7 years of age, she obtained an intelligence quotient of 40. Cranial magnetic resonance imaging, at 9 years of age, did not reveal any abnormalities. On recent assessment, at 11 years of age, her motor skills had improved significantly but she still had speech and social interaction difficulties, as well as learning disability. From the point of view of phenotype, she has been growing proportionately with age without any strong phenotypic features. As can be observed in Figure 1, she presents only slight facial dysmorphism, having a long face with a prominent chin, broad forehead, and a broad, large mouth with widely spaced upper front teeth and slightly large and detached ears.

### 2.1. Genetic Analysis

With written informed consent from the parents, we carried out initial genetic studies, including karyotyping and molecular analysis of* MECP2* by means of MLPAs and Sanger sequencing. After that, we decided to perform array comparative genomic hybridization (array CGH) in samples from the patient and her parents, and results were confirmed with quantitative reverse-transcription polymerase chain reaction (qRT-PCR) assays, using applied biosystems real-time PCR instruments and software.

Firstly, DNA was purified from peripheral blood according to standard protocols and a Perkin Elmer CGX Oligo Array 8x60K was used to perform genome-wide copy number analysis. This microarray covers over 245 cytogenetically relevant regions, as well as genes involved in development, pericentromeric regions, and subtelomeres. The Agilent SureScan microarray scanner and Agilent Feature Extraction 11.0.1.1 software were used according to the manufacturer's instructions. Results were analyzed with CytoGenomics v.2.7 (Agilent) and Genoglyphix (Signature Genomics) software.

To validate the results, qRT-PCR was performed in a final volume of 20.0 *μ*L using SYBR Green real-time PCR Master Mix Kit and the 7900HT fast real-time PCR System (both from Life Technologies), in accordance with the manufacturer's instructions. Three pairs of primers were designed using Primer 3 Plus software.

### 2.2. Genetic Results

**Figure 2:**
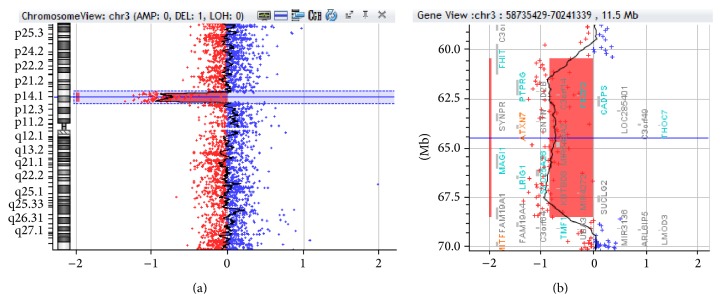
The array comparative genomic hybridization (CGH) profile of chromosome 3 showing an interstitial deletion. (a) View of chromosome 3 and (b) the enlarged view of the rearrangement as generated by CytoGenomics v.2.7 (Agilent Technologies). The deletion breakpoint was between 60.461.316 and 68.515.453 (3p14.2-p14.1). The size of the deletion was ~8 Mb.

The karyotype was normal, as was the* MECP2* gene. However, array CGH analysis revealed an 8 Mb proximal deletion at 3p14.2-p14.1, from position 60.461.316 to 68.515.453 (Figure 2) (GRCh37/hg19). Among the genes mapped to this region, six are known or believed to have a role in neurodevelopment:* FEZF2* (OMIM# 607414),* CADPS* (OMIM# 604667),* SYNPR* (no OMIM entry),* TXN7* (OMM# 607640),* PRICKLE2* (OMIM# 608501), and* MAGI1* (OMIM# 602625). The deletion was confirmed to be de novo, on the basis of a comparative study with the parental DNA by qRT-PCR (Table 1).

## 3. Discussion

Several microdeletions and microduplications mapped to 3p have been identified in patients with developmental disorders, autistic features, and/or global developmental delay. However, the lack of characteristic facial dysmorphisms or other distinct external features in these patients makes it very difficult to diagnose without array CGH technology. Nevertheless, it is known that a correct diagnosis is important to estimate recurrence risk for genetic counseling and may also play an essential role in improving the clinical management of these patients. Here, we have reported a case of a de novo 8 Mb microdeletion of 3p14 in an 11-year-old girl with speech and social interaction difficulties, as well as mild facial dysmorphisms.

**Figure 3:**
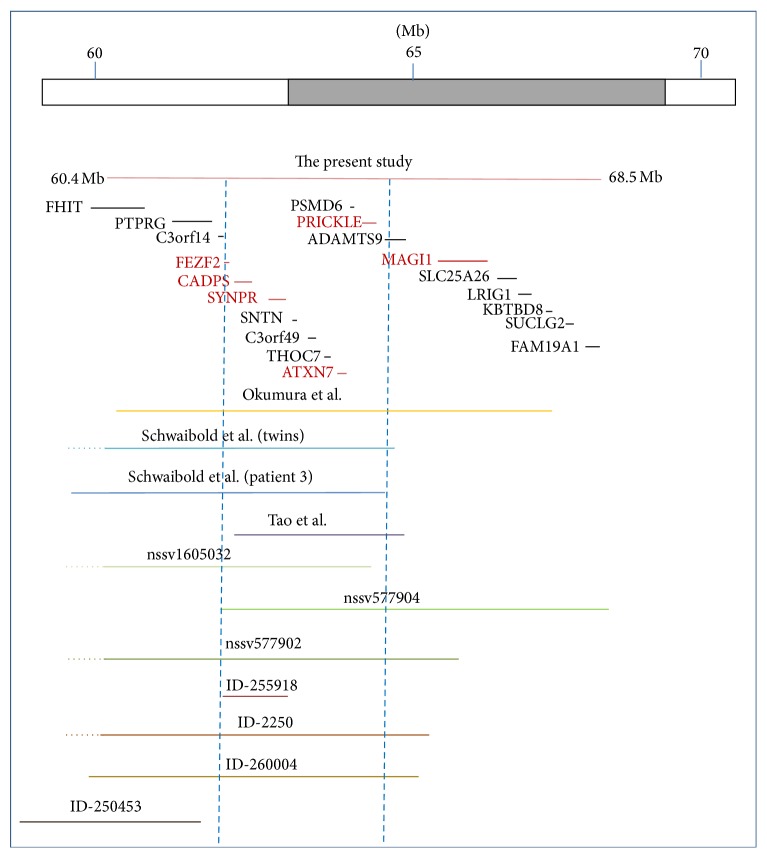
Schematic representation of the 3p14 deletions. The orange line represents the deletion in our patient. The deletions found by Okumura et al., Schwaibold et al., and Tao et al. are represented by lines in yellow, blue, and purple, respectively. The green and brown lines are deletions described previously in ISCA Consortium and DECIPHER databases, respectively. The thin red and black horizontal lines indicate the genes that are located in the 3p14 deleted region, the red ones being those that might be responsible for the phenotypic features given their known biological functions. The blue vertical dashed lines indicate the region in which the candidate genes are located and the overlapping deleted regions in 10 of the 11 cases.

Table 2 summarizes the clinical features of patients reported previously [9–11], as well as seven other 3p14 carriers listed with full clinical descriptions in DECIPHER. The DNA sequence between 60.461.316 and 68.515.453 points in chromosome 3 (Figure 3) contains 19 genes, 6 of which encode proteins that could be responsible for the phenotypes observed in these patients. These candidate genes are* FEZF2*,* CADPS, SYNPR, ATXN7, PRICKLE2,* and* MAGI1*. The* FEZF2* gene encodes a transcription factor that is required for the specification of corticospinal neuron identity and connectivity [12, 13], and the* SYNPR* gene encodes a protein that is an integral membrane component of synaptic vesicles [14]. The* CADPS* gene is expressed in the fetal and adult brain and it is an essential regulator of synaptic vesicle and large dense core vesicle priming in mammalian neurons and neuroendocrine cells [15]. Expansions in* ATXN7 *cause spinocerebellar ataxia type 7, but the role of other kinds of mutations (nonsynonymous substitutions or deletions) in this gene in nervous system disorders is not yet well understood [9]. The fifth gene is* PRICKLE2*, which encodes a postsynaptic Wnt/planar cell polarity pathway component required for the normal development of synapses [16] and whose disruption in mouse hippocampal neurons leads to reductions in dendrite branching, synapse number, and postsynaptic density. It has recently been shown that disruption in* PRICKLE2* is associated with behavioral abnormalities including altered social interaction, learning abnormalities, and behavioral inflexibility [17]. On the other hand, though Okumura et al. [9] propose* PRICKLE2* as the most likely causative gene of autistic features observed in their cases, this is not consistent with earlier findings by other authors [11, 18]. Finally,* MAGI1* is a protein of membrane-associated guanylate kinase (*MAGUK*) complexes that act as key scaffolds in surface complexes containing receptors, adhesion proteins, and various signaling molecules, playing key roles in cell-to-cell communication.* MAGUK* proteins are present in neuronal synapses and they help to organize the postsynaptic structure via associations with other scaffolding proteins [19]. Previous studies have demonstrated an association of* MAGI1* copy number variation with bipolar affective disorder [20].

Regarding the study conducted by Schwaibold et al. [10], the deletion found in monozygotic twins was around 6.32 Mb long, with breakpoints between 3p14.1 and 3p14.3 (58.244.794–64.571.699), and in the adult patient, the deletion was approximately 4.76 Mb long, with breakpoints between 3p14.1 and 3p14.2 (59.443.171–64.162.112). The three patients had very similar features, namely, intellectual disabilities, gross-motor delay, slight facial dysmorphism, nonexpressive language, and autistic features, although the adult started to show friendly behavior in adolescence. The phenotypes of the cases reported by Tao et al. [11] with microdeletions from 62.665.527 to 64.890.116 and by Okumura et al. [9] with breakpoints between 60.472.496 and 67.385.119 were also quite similar.

Although the deletion found in our patient is considerably longer and extends further toward the centromere, especially compared to deletions described by Schwaibold et al. [10] and Tao et al. [11], respectively, the phenotypic features are quite similar to those in the previously described patients with deletions in 3p14. Moreover, none of the additional genes deleted in our patient seems to play an important role in brain development; with the exception of MAGI1, which was also deleted in Okumura et al.'s twins [9] (Figure 3). Therefore, we believe that our findings support the conclusions of previous authors who have indicated that the candidate gene(s) for these common features may well be among those located in the region distal to the centromere, between breakpoints 62.3 and 64.5 Mb (the left part of the deletion in Figure 3), and a combination of genes in this region involved in brain or cognitive development might be responsible. Apart from the patients reported in the literature [9–11, 18] and the cases mentioned by Schwaibold et al. [10] which are reported in the DECIPHER database, we are aware of another three patients, reported in the International Standards for Cytogenomic Arrays (ISCA) Consortium database with their phenotypes, that have overlapping deletions in 3p14 (Figure 3). All these patients have similar features and also showed global developmental delay.

## 4. Conclusion

Our results support the hypothesis that a novel 3p14.2 core region in 3p proximal deletions is associated with neurodevelopmental disorders. We were not able to identify a single gene responsible for the phenotypes associated with microdeletions in 3p14, but we rather believe that several candidate genes located in this region could be the cause of these disorders. In consequence, we consider it very important to report new cases with overlapping deletions in the 3p segment to more precisely identify the genotype-phenotype correlation.

The characteristic lack of external features in these patients makes it difficult to reach a correct diagnosis without high-resolution molecular cytogenetic techniques such as array CGH.

## Figures and Tables

**Table 1:** Validation with quantitative reverse-transcription polymerase chain reaction (qRT-PCR) assays. This table shows the values obtained by means of qRT-PCR in the patient analyzed and her mother. Two amplicons were amplified by qRT-PCR; they were located in the deleted region (3p14.2-p14.1), in the *FAM19A1* and *ATXN7* genes. The control gene used to normalize the data was *RPP30*, located at 10q23.31. The 2^−ΔΔCt±SD^ values of the patient were less than half the values of her mother, who was used as the normal control.

	2^−ΔΔCt±SD^	
	Patient	Mother
FAM19A1	0.480	1.06
ATXN7	0.450	1.06

ΔΔCt = (Ct_target_ − Ct_Ref_)DNA_test_ − (Ct_target_ − Ct_Ref_)DNA_Ref_.

SD_DNA test_ = (sd1^2^ + sd2^2^)^1/2^.

2^−ΔΔCt±SD^.

**Table 2:** Summary of patients with overlapping deletions in 3p14.

Patient	Deletion breakpoints	Developmental delay/ID	Speech impairment	Autistic features	Ear anomalies	Facial dysmorphisms	Limb anomalies	Other distinctive features
Present Study	60.461.316–68.515.453	Severe	Yes	Yes, lack of a social smile, difficulties with eye contact, stereotypic movements	Slightly large and detached ears	Long face, prominent chin, broad forehead, wide mouth, widely spaced upper front teeth	None	motor developmental delay

Okumura et al., [9] Twins A/B	60.472.496–67.385.119	Severe	No expressive language until the last follow-up at 49 months of age.	Yes	Low-set, posterior rotated	Arched down-slanting eyebrow, prominent forehead, epicanthic folds, micrognathia, hypertelorism, broad nasal brige, short philtrum	Camptodactyly	Twin 2: intestinal malrotation, ventriculomegaly

Schwaibold et al., [10] Twins 1/2	58.224.794–64.571.699	Extend no specified	Undirected double syllables at 2 10/12 years of age	Yes (stereotypic movements)	Low-set, slightly posterior rotated	Arched, downslanting eyebrows, positional plagiocephaly, Twin B: cowlicks	Twin B: Thick left thumb with sites for two nails	Severe feeding problems, small stature, Twin B: hydrocephalus, hypoplasia of corpus callosum

Schwaibold et al., [10] Patient 3	59.443.171–64.162.112	Extend no specified	No active speech, he follows simple orders at 18 years of age	Yes, in his adolescence he started to develop eye contact	None	Broad mouth, prominent chin, widely spaced teeth, deep-set eyes, long slender face, flat occiput	High tonicity in lower limbs	Brain anomalies in MRI

Tao et al., [11] Patient 6	62.665.527–64.890.116	Severe	N/M	Yes	N/M	N/M	N/M	Epilepsy

D2250	54.452.525–65.609.348	Extend no specified	None	N/M	Extend no specified	Wide mouth, high palate, broad forehead, epicanthus, thick eyebrows	Camptodactyly, talipes equinovalgus, valgus, ulnar deviation of hands	High palate, strabismus

D250453	58.717.185–61.696.115	Extend no specified	Extend no specified	N/M	Low-set, posterior rotated, abnormality of the pinna	Low anterior hairline, mandibular prognathism, widely spaced teeth	N/M	Nevi, lentigines

D255918	62.749.576–63.021.934	Extend no specified	N/M	N/M	N/M	Plagiocephaly	2-3 toe syndactyly	Scrotal hypoplasia

D260004	59.813.606–65.296.648	Extend no specified	Severe	N/M	Low-set, posterior rotated	Prominent forehead/fontal bossing	Clinodactyly	None

nssv1605032	57.416.265–64.870197	Extend no specified	N/M	N/M	N/M	N/M	N/M	Muscular hypotonia

nssv577904	61.956.521–68.514.983	Extend no specified	N/M	N/M	N/M	N/M	N/M	N/M

nssv577902	54.079.045–66.046.136	Extend no specified	N/M	N/M	N/M	N/M	N/M	Failure to thrive, microcephaly

N/M: Not mentioned.
